# Barriers and facilitators of physical activity among school attending adolescents in Lagos State, Nigeria: A qualitative study exploring views and experiences of decision‐makers in secondary schools

**DOI:** 10.1002/hsr2.997

**Published:** 2022-12-20

**Authors:** Busola Adebusoye, Jo Leonardi‐Bee, Revati Phalkey, Kaushik Chattopadhyay

**Affiliations:** ^1^ Nottingham Centre for Public Health and Epidemiology, School of Medicine, City Hospital Campus University of Nottingham Nottingham UK; ^2^ The Nottingham Centre for Evidence‐Based Healthcare University of Nottingham Nottingham UK; ^3^ Centre for Climate Change and Health Security UK Health Security Agency London UK

**Keywords:** adolescents, barriers, facilitators, Nigeria, physical activity, schools

## Abstract

**Background and Aims:**

Schools represent a unique setting for promoting lifelong physical activity during critical development stages of life. Opportunities for in‐school physical activity largely depend upon school‐level policies, practices, and administrative support. A significant information gap exists on the factors influencing adolescents' participation in school‐based physical activity programs in Nigeria. This study aimed to identify and explore the barriers and facilitators of physical activity in school‐attending adolescents in Lagos State, Nigeria.

**Methods:**

A qualitative study, using semistructured interviews, was conducted to explore the views and experiences of 21 decision‐makers who were responsible for planning the physical and health education curriculum in secondary schools in Lagos State, Nigeria. Data were analyzed using the thematic analysis framework.

**Results:**

Eight themes were identified and explored. The barriers were (i) students' characteristics, (ii) parental objections, (iii) no prioritization of physical activity, (iv) insufficient resources, and (v) challenges with schools' initiatives. The facilitators were (vi) students' interests, (vii) students' awareness of benefits, and (viii) schools' initiatives.

**Conclusion:**

Our study findings can help in designing interventions to increase physical activity among school‐attending adolescents in Lagos, Nigeria.

## 1 INTRODUCTION

Physical activity offers immense benefits to adolescents, such as helping them develop and maintain healthy musculoskeletal tissues, cardiovascular systems, and body weight.^1^ Physical activity improves their mental health by reducing depression, anxiety, and stress.^1^ It positively impacts students' academic achievements and overall quality of life.^2^, ^3^ Physical activity also assists in their social development by providing opportunities for self‐expression, improving self‐confidence, social interactions, and integration.^4^

Despite the benefits accrued from being physically active; only approximately 19% of the global school‐going adolescents reach the level recommended by the World Health Organization (WHO); however, this is even lower in sub‐Saharan Africa, where only 13.8% of school adolescents are reported to reach the recommended level.^5^ The WHO recommends that schools should provide quality physical and health education that supports adolescents to develop healthy behavior patterns that will keep them physically active throughout their lives.^1^ In keeping with this recommendation, Nigeria developed a National School Health Policy in 2006, which proposes practising physical activities for the health, academic, and remediable problems (e.g., sleep problems, substance use) of school adolescents.^6^ However, very little is known on the implementation of this policy in schools.^7^

Current evidence suggests that the overall physical activity levels in Nigerian school adolescents are low, ranging from 5% to 37%.^8^, ^9^, ^10^ These studies have reported some sociodemographic factors such as religion, parents, socioeconomic status, number of children in the family, motivation, self‐efficacy, age, and weight status as factors associated with physical activity. Studies conducted in Lagos State have shown that the type of schools adolescents attend can impact their participation in physical activities.^11^ There is also evidence of increased participation in physical activities of adolescents in schools following a professional development training program for teachers to promote physical activity in adolescents in Lagos State.^12^

Lagos State occupies a unique position in Nigeria as the country's largest urban area, and it has over a thousand secondary schools to accommodate its teeming adolescent population.^13^ With urbanization comes the inequitable distribution of resources that impact physical activity.^14^ Physical activity in Lagos particularly for the lower socioeconomic group, which comprises most of its residents, is undertaken in unsupportive and potentially harmful environments while navigating dangers such as air pollution and road traffic injury.^15^

Schools, however, represent a unique setting for promoting lifelong physical activity during critical development stages of life.^16^ Opportunities for in‐school physical activity largely depend upon school‐level policies, practices, and administrative support.^17^ A significant information gap exists on the factors influencing adolescents' participation in school‐based physical activity programs in Nigeria.^7^ No study has considered the experiences and views of key decision‐makers in schools who are responsible for the management of schools in Lagos State, Nigeria. Interventions to promote physical activity in adolescents should be informed by knowledge of the factors that influence it.^18^ Therefore, this study identified and explored the views and experiences of school decision‐makers on the barriers and facilitators of physical activity among school‐attending adolescents in Lagos State, Nigeria.

## 2 METHODS

### 2.1 Study design

A qualitative study was conducted to address the aim of the study and the study was reported according to the COREQ guidelines of reporting qualitative studies.^19^

### 2.2 Study participants

The study participants were decision‐makers, such as principals, vice‐principals, and district and state officials in the Lagos State Ministry of Education, responsible for planning the physical and health education curriculum of secondary schools. They were from schools that were selected using stratified random sampling. In the first stage, schools were stratified by local government area and then by school type using the master list accessed from the official internet portal of all schools in Lagos State.^20^ School officials who were not responsible for the curriculum planning were excluded.

### 2.3 Recruitment

Decision‐makers in one of the six districts of Education in Lagos State, which has over 150 public and private secondary schools, were contacted through their offices. They were briefed about the research aims and their willingness to participate; they were given a participant information sheet that contained detailed information about the purpose of the study, why they were approached, and the confidentiality of the data. The participant was given an informed consent form signed by both the lead researcher and the participant. Then, the place, date, and time to conduct the interview were scheduled.

### 2.4 Interview guide

The interview guide was developed using previous literature that has identified and explored the barriers and facilitators of physical activity in school adolescents among decision‐makers in schools.^21^, ^22^ The interview guide had 10 questions which included questions asking: Is physical and health education offered in your school as a subject? Could you please tell me about the physical activity participation of the students in your school? Could you please tell me about students' physical activity levels in your school? Could you please share your thoughts on the things preventing students from getting more exercise in school? What things could the school do to make it easier for students to get more physical activity at school? If physical activity opportunities are improved, how do you think this might affect the current schools' curriculum? How inclusive will you describe the physical activities offered in your school? Could you please share your thoughts on how culturally sensitive issues (religion, culture) influence physical activity participation among the students? How will you describe teachers' motivation to be involved during physical activities in your school? The interview guide had questions that allowed probing of participants' responses. The interview guide was piloted with one school principal, and the transcript was included in the data as no changes were made to the interview guide.

### 2.5 Data collection

A trained qualitative researcher conducted the semistructured interviews. Face‐to‐face interviews in the participants' places of work (schools/offices) were planned for all the interviews, but due to the COVID‐19 pandemic, 12 interviews were conducted over the phone. The interviews were recorded with permission using a digital recorder. The interviews were conducted from March to September 2020 in English since it is the country's official language.

### 2.6 Data analysis

The lead researcher transcribed three of the interviews verbatim, and the remaining 18 were transcribed verbatim by a professional transcriber after signing a nondisclosure agreement. All identifiable information was removed. All the transcripts were compared against the recordings for accuracy, and any discrepancies were corrected. The lead researcher read the transcripts several times to become familiar with the data. Data were analyzed using the deductive thematic analysis framework of Braun and Clarke.^23^ The first interview transcript was analyzed by hand by the lead researcher to generate the initial codes. Subsequent transcripts were analyzed using NVivo 12 (QSR International Ltd). Codes were organized into overarching categories, after which themes and subthemes were assigned. The lead researcher reviewed themes to ensure that they were distinct and not overlapping. The process was continuously discussed with senior study authors to refine the themes. The themes were further considered in relation to the whole data set to ensure they accurately reflected the data set. Anonymized quotes annotated by role and type of school are presented to support the defined themes. During the analysis, the lead researcher referred to her reflexivity notes to examine how her beliefs and judgments could have influenced the findings.^24^ For the reflexivity notes, the lead researcher was aware of her gender and her experience of the physical and health education classes she attended in secondary school; therefore, the interview guide prepared beforehand helped to mitigate the effects of the researcher's bias in the interviews.

## 3 RESULTS

Twenty‐one school decision‐makers were interviewed. Ten (six principals and four vice‐principals) were from private schools, and eight (two principals and six vice‐principals) were from public schools. The remaining three participants were district and state officials in the Lagos State Ministry of Education. Fourteen participants were males. The semistructured interviews ranged from 8 to 50 min (mean duration of 20 min).

## 4 THEMES

A total of eight themes categorized into student‐, parent‐, and school‐related barriers and facilitators were identified and explored. Five were barriers, and three were facilitators. The barriers were (i) students' characteristics, (ii) parental objections, (iii) no prioritization of physical activity, (iv) insufficient resources, and (v) challenges with schools' initiatives. The facilitators were (vi) students' interest, (vii) students' awareness of benefits, and (viii) schools' initiatives. Table 1 shows the themes and subthemes of the barriers and facilitators.

**Table 1:** Themes and subthemes

Themes	Subthemes
Barriers	
Student‐related barriers	
1. Students' characteristics	1. Physical disabilities or health status
	2. Body image concerns
	3. Little or no interest in physical activities
	4. Belief that physical activities were more suited for boys
	5. Boys' monopoly of school playgrounds
	6. Girls' religious norms, such as not wearing shorts or trousers for physical activities
Parent‐related barriers	
1. Parental objections	1. Physical activities will lead to less time for academic activities
	2. Physical activities will lead to pregnancy problems in daughters
	3. Physical activities will lead to injuries
School‐related barriers	
1. No prioritization of physical activity	1. Physical and health education was offered only in junior classes
	2. Emphasis on students' academic engagements
	3. Physical and health education is more theoretical than practical
2. Insufficient resources	1. Lack of physical and health education teachers
	2. Lack of financial resources for facilities and equipment
3. Challenges with schools' initiatives	1. Heavy traffic made it difficult to transport students to community facilities
	2. Students engaged in brawls during interhouse sports competitions
Facilitators	
Student‐related facilitators	
1. Students' interest	1. Students enjoyed physical activities
	2. Students pursued individual physical activity interests outside of the school setting
2. Students' awareness of benefits	1. Students' awareness that physical activities make them fit
	2. Students' awareness of the financial incentives from professional sports
School‐related facilitators	
1. Schools' initiatives	1. Organizing weekly physical activity sessions by the schools during school hours
	2. Organizing the annual sports competition by the school
	3. Schools' key decision‐makers responding to students' concerns
	4. Sensitizing students and parents on the benefits of physical activity in adolescents
	5. Enlisting third‐party organizations to engage students in physical activities

### 4.1 Barriers

#### 4.1.1 Student‐related barriers

##### Students' characteristics

This theme describes how students' attributes, such as physical disabilities, health status, or sex differences, prevented them from being physically active. This theme comprises six subthemes.

“Although some students have challenges, like those with SS (Sickle cell anemia),… we normally exempt them because of their health status.” [District Official 2, District]

It was also noted that such students were avoided by their peers during physical activities even if they showed interest.

“We have some students that are SS, and because some students want to play safe, once those SS get to the field, you will hear others shouting, “I'm not playing ball with you,” they only just want to play safe and not get into any trouble.” [Principal, Private School]

Two participants mentioned that overweight students were usually not interested in physical activity.

“Yeah, of course, you know that some girls are lazy, so you force them to do that… and probably sometimes fat ones; they are not ready to lift their legs.*”* [Principal, Private School]

One of the participants commented that girls perceived that physical activity was not meant for them.

“We notice with the girls, they are too self‐conscious of themselves, and they have this idea of whether it is cool or not… is it cool to run about as boys do?” [Principal, Private School]

There were also some comments regarding the poor participation of girls in physical activity, due to boys' monopolization of the schools' playgrounds, particularly for football.

“But you know how boys do now? Boys play football every day, and sometimes when the girls feel like they want to play, they approach me or tell somebody else that we should please chase the boys away; they want to play.” [Principal, Private School]

Some participants commented on how religious norms, like the accepted mode of dressing, affected girls' participation in physical activity.

“Some people out of religion say they don't want to wear shorts, or some don't want to expose their legs.” [Vice‐Principal, Public School]

### 4.2 Parent‐related barriers

#### 4.2.1 Parental objections

Many participants commented that some parents felt that involvement in physical activity affected their children's academic performance. Hence, they discouraged their children from participating in physical activities.

“Then parents attitude sometimes because some parents believe that they should not do anything in school apart from the cognitive aspects. You see the children being willing sometimes, but because of what their parents say, like, “I have not sent you there to go and play football or run; you are supposed to go there and count all the As that you can in the academic sphere.” [Principal, Private School]

Some participants also commented on the parents' views on what physical activity is permitted, particularly for the girl child, and how physical activity might delay their daughters' development, particularly concerning pregnancy.

“Because some of them will come with the fact that it affects girls' development, that girls may not be able to give birth, so with the parents, we just said we would pull it down, and we have been able to do that over time.” [Principal, Private School]

There were also comments about parents' fears that their children might become injured if they participate in physical activities.

“Do you know where they can get to? I will be spending on this child, and he will go and get a broken leg.” [Principal, Public School]

### 4.3 School‐related barriers

#### 4.3.1 No prioritization of physical activity

This theme describes how students' engagement in physical activity was not taken as a priority in schools. It comprises three subthemes. All participants confirmed that physical and health education is offered as a compulsory subject in the junior class but optional or not available in the senior classes. Some reasons, such as the subject not being a prerequisite for most of the courses in the university, or students can only offer a limited number of subjects for qualifying exams or no scheduled time for it on the schools' timetables, particularly for senior secondary students, were cited.

“Yes, it is offered only in the junior class between grade 7 and grade 9.” [Principal, Private School]

Some participants commented that more emphasis was on the student's academic study than physical activity. This is caused by the scheme of work in schools which many of our participants considered to be voluminous. They commented that the overloaded scheme of work made it difficult to allocate time for students' engagement in physical activities. Even sometimes, teachers use some allotted time for physical activities to teach the students some parts of their subjects whenever they cannot meet their expected teaching objectives.

“It is the overloaded scheme of work, that is a major one, they're overloaded and the normal subjects they are offering to me, it's more than usual, 13 or 14, which will be reduced to about eight, maximum of eight when they are writing their exam.” [Vice‐Principal, Private School]

Some participants said that the approach to the subject is more theoretical and involves little or no time for practice. The inclusion of practice if at all, is left to the teacher's discretion.

“Even… when you are looking at the syllabus in the junior secondary school, we don't have much practical. We have much of theories in the scheme.*”* [District Official 1, District]

#### 4.3.2 Insufficient resources

This theme describes the limited resources that were barriers to physical activity. This theme was recurrent for most participants. Concerning human resources, some of our participants said they had no teachers to teach the subject or very few teachers to effectively cater to the student population.

“The lack of trainers, because we have over 1000 students in this school and only one physical health education teacher. So, for just one person to take over 1000 students, it's too much. So, I would say we need more trainers.” [Vice‐Principal, Public School]

Concerning financial resources, some of the participants commented that due to a lack of funds, they didn't have space, facilities, or equipment that could engender various activities.

“And you know this is an urban school, and one of the major problems in an urban school is space. So, it's not a school with much space where you can assign much space for different games and physical activities.” [Principal, Private School]

#### 4.3.3 Challenges with schools' initiatives

This theme describes how different circumstances have undermined the schools' opportunities to encourage physical activity among the students. One of our participants mentioned that the traffic situation in Lagos State had mitigated their use of community facilities within the school's environs, reducing the number of times they could go there.

“You may decide to spend this one hour to go to the stadium, and before you know it, you spend three hours going to the stadium and coming back. So, what we do is that occasionally, we go outside the school to use community facilities.” [Principal, Private School]

Another participant mentioned how the interhouse sports they do are usually limited because students engaged in brawls because of the conflicting issues among them.

“Probably yes, during the inter‐house sports,… because you know, at the adolescent age, the truancy tendency is there… so, some people use the sporting activities as a time to revenge, so those things deter us.” [Vice‐Principal, Public School]

### 4.4 Facilitators

#### 4.4.1 Student‐related facilitators

##### Students' interest

Some of our participants commented that many students were enthusiastic about physical activities and embraced such opportunities presented to them.

“Being youths, they are always willing to use up their energy, so whenever the school gives them the opportunity, they fully participate.” [Vice‐Principal, Public School]

Some participants commented on students who explored their physical activity interests outside school settings. They further reported that the schools supported them to partake in external competitions whenever there was a need.

“We have one particular girl… representing Lagos State in handball, so we have one or two students like that who participate in physical activity, but they do it outside. When there is a need for that competition, they normally seek permission to engage in it outside.” [Vice‐Principal, Public School]

#### 4.4.2 Students' awareness of benefits

Some participants reported that students' awareness of the benefits (health and financial benefits) of physical activity kept them engaged in physical activities. Benefits such as having good shape, particularly for girls, and the possibility of getting into professional sports, which have immense financial rewards, were motivations for boys and their parents.

“But I also notice that some of these parents who are poor, so to say, encourage their children to participate, especially in football, for financial reasons; with the hope that they will make great footballers and earn some income.” [Principal, Private School]

#### 4.4.3 School‐related facilitators

##### Schools' initiatives

This theme describes the different actions that different schools have taken to encourage physical activity in their schools. These initiatives include organizing weekly physical activities during school hours, organizing annual interhouse sports competitions, school decision‐makers responding to students' concerns, sensitization of parents, and schools allowing external organizations to engage their students in physical activities. Five participants reported scheduled times for weekly opportunities for their students to engage in physical activities.

“Like I told you on Friday, it's a must for everybody. On the other days, we can just leave anybody that's just willing to play… But on Friday it is a must for everybody” [Principal, Private School]

Other participants talked about having an annual sports competition where students participate in physical activities both during the time of the competition and in the build‐up to the competition.

“then once every year or so we do what we call our inter‐house sports… that gives room to all the students now to participate but that happens once every year.” [Principal, Private School]

Also, some participants shared their experiences of responding to the concerns and interests of the students. They responded to the needs of students who for religious reasons do not want to wear some types of clothes or for those who had interests in other activities.

“But what we do is those who don't want the shorts, we sew… with a flap in front and at the back to cover the gap in between the legs, so that's what we do.” [Vice‐Principal, Public School]

Some participants commented on schools sensitizing the students and the parents on the benefits of physical activity to students.

“I think it is very important to sensitize not just the students but the parents… and tell them how important physical activity is.” [Principal, Private School]

Finally, five participants recounted their experiences of enlisting organizations such as corporate organizations, nongovernmental organizations, and old students' associations to provide resources (both human and facilities/equipment) that engaged the students in physical activities.

“We have somebody who comes, not a teacher but he is hired, he comes here, and they take both the students and of course teachers, especially the males, they go there (community field). They do some jogging; some do a kind of dancing.” [Vice‐Principal, Private School]

## 5 DISCUSSION

This study identified and explored five barriers and three facilitators to physical activity in school‐attending adolescents among decision‐makers in secondary schools in Lagos State, Nigeria. These were categorized into student‐, parent‐, and school‐related barriers and facilitators.

Our study found students' poor participation in physical activities due to physical disabilities, body image concerns, health status, or lack of interest. Our finding of the exclusion of adolescents with physical disabilities and health challenges by their peers is similar to the finding reported in a scoping review which explored the barriers and facilitators to participation in adolescents in low‐ and middle‐income countries. They reported that adolescents with physical disabilities were excluded by their peers and this made them feel embarrassed at appearing physically inept.^25^ In addition, lack of interest in physical activity and laziness were also reported as barriers in a study that was conducted in Morocco, North Africa.^22^

Our study's finding regarding overweight students not participating in physical activity is similar to a study that showed that overweight students were discriminated against for negative expectations about their physical ability by their peers or teachers. This was reported to affect their participation in physical activities.^26^ Existing evidence from a narrative review which included data from 1983 to 2013 reported that about 1.0%−8.6% of Nigerian adolescents were overweight.^27^ Recent studies have shown a prevalence of about 5.8%−9.7%.^11^, ^28^ A continuous increase in the prevalence of overweight could further compound this barrier to physical activity in the future, creating a vicious cycle of both increasing adolescence overweight and decreasing physical activity levels. This calls for targeted interventions of behavioral modifications such as diet and physical activity, which have shown to have moderate quality evidence for the lowering of body weights in adolescents.^29^

Our study's finding of boys being more interested in physical activities than girls and girls being constrained by cultural norms was supported by a study which was conducted in Canada and India. The study reported that girls were less likely than boys to be interested in physical activity, with girls' participation in India further limited by societal restrictions.^30^ This was also found in other studies conducted in Nigeria and Morocco, where girls were prevented from participating in sporting activities due to cultural factors such as mode of dressing,^22^ or misconceptions of the impact of physical activity.^7^ A further study reported that female teenagers dropped out of sports they enjoyed because they felt that the sports were masculinizing their bodies.^26^

Our finding of parents preventing their female children from participating in physical activity over concerns that they will not be able to get pregnant was also similar to a finding reported in another study in Nigeria.^7^ Similarly, a further study in South Africa reported that parents prevented their female children from engaging in physical activities to keep them safe from sexual violence.^31^ In addition, our finding of parental fears over their children getting injured during physical activities was supported by a study in Bangladesh, where such parental fears led to changes to the activities offered in the physical education curricula.^32^

Our finding that physical activity was not prioritized in schools is similar to the findings from studies conducted in Nigeria and South Africa, where physical and health education classes were more theoretical than practical^7^, ^31^; or schools had no classes allocated to physical and health education.^33^ Also, our finding of physical activity conflicting with the academic study is similar to that reported in a South African study, where students in the senior classes of secondary school were told to use their time to study rather than get involved in physical activity.^34^ This finding persisted in other studies conducted in Morocco and in Nigeria where it was reported that some teachers and principals see physical and health education as a waste of time and suggest the time should be devoted to other academic subjects.^7^, ^22^ Our finding of limited human resources inhibiting physical activity is similar to what was reported in another study conducted in Nigeria, where participants reported a declining number of trained physical and health education teachers which they argued might be due to limited opportunities for continuing education and professional development for physical and health education teachers compared to other subjects like Mathematics and English that were deemed to be of higher relevance. Also, the study reported a lack of financial resources for facilities or equipment.^7^ Schools' facilities such as large playgrounds are associated with physical activity in adolescents.^35^, ^36^ Finally, for school‐related barriers, our finding of students engaging in brawls after interhouse sports competitions was also noted in another study in Nigeria in which it was reported that interhouse sports were more frequently practiced in private schools compared to public schools due to the aftermath crisis associated with the failure of losing teams to accept defeat in public schools.^7^

Our finding of students participating in physical activity because of their interests and health benefits is similar to what was reported in studies conducted in South Africa and Morocco where adolescents were reported to be inclined to be physically active because of the health benefits associated with it and also for enjoyment.^22^, ^31^ In addition, we found a similar finding with students participating in physical activities for financial gains in Nigeria and South Africa. Adolescents were incentivized to take part in sporting competitions by the prospect of cash prizes.^7^, ^31^, ^37^

Schools' initiatives such as scheduling weekly physical activities and organizing annual interhouse sports competitions which provided opportunities for students' participation in physical activities have been cited as reasons for physical activity participation in students.^7^, ^22^ About 15 participants said they have annual interhouse sports competitions which provided opportunities for students' participation in physical activities. It, however, appears that because of the competitive nature of the activities, only students with physical activity prowess would benefit from such competitions. While sporting competitions are good for the identification and nurturing of innate talents, these might mask the importance of schools' physical activity which should be promoted with a respectful and helpful attitude avoiding attitudes of superiority.^26^

Two of our participants reported having responded to the concerns of their students which suggests social support from teachers. Social support from teachers have been shown to elicit positive responses from students encouraging them to be physically active.^22^, ^38^, ^39^ One of our participants commented that girls responded positively to activities that involved dance, this finding is corroborated by other studies which showed that dance classes have been shown to provide valuable opportunities for adolescent girls to be physically active.^31^, ^34^, ^40^

The initiative of enlisting third‐party organizations to engage the students in physical activity is in line with the United Nations Educational, Scientific and Cultural Organization's quality physical education guidelines. The guidelines encourage the development of partnerships between schools and community‐based sports organizations. There is evidence that students are more likely to be physically active in schools where there is a well‐established school‐community partnership.^41^

When comparing our findings to high‐income countries, there are some similarities in the barriers like limited resources. For example, in a study that was conducted in the United States of America which compared the barriers and facilitators between urban and rural youths; the study reported limited finances as a barrier where the parents needed finances to either pay to use the facilities for urban youths or for transportation to use the facilities for the rural youths.^21^ Another similar barrier was the conflict with academic studies where adolescents complained of having to spend much of their time on school homework and did not have time to be involved in physical activities in the United Kingdom and United States of America.^42^, ^43^, ^44^

Finally, our study shows that the senior (last 3 years of secondary school) students do not usually offer physical activity as a subject. The Lagos State Government should consider scheduling physical activity for senior students, whom our study has identified to lack a structured time for physical education classes. Additionally, stakeholders should ensure that the scheduled classes, for all students, guarantee students' participation in physical activity as is done in other parts of the world and not just the theoretical aspect.^45^, ^46^

## 6 STRENGTHS AND WEAKNESSES

To our knowledge, this is the first qualitative study that identifies and explores the barriers and facilitators of physical activity among school adolescents in Lagos State, Nigeria. One of the strengths of this study is the diversity of the interviewed decision‐makers, which made us explore different views and experiences. Interviewing private and public school participants helped generate transferrable insights into both schools in Lagos State, Nigeria. Also, the semistructured interview allowed the lead researcher to delve deeply into the participants' responses, thereby generating more insights into the research aims. We reached data saturation of findings at the 14th interview; we, however, continued the interviews to ensure that we did not miss any unique information.

A potential weakness is that the average length of the interviews (20 min) seemed insufficient to explore views and experiences deeply. However, this is unlikely to have affected the findings since the same interview guide was used. Also, the similarity of our findings to those from other similar studies shows the richness of the data generated by our interviews. We acknowledge that our population has demographics and cultural characteristics which do not allow generalizability to be inferred, but we have addressed this by providing a comprehensive description of the context of the study.

## 7 CONCLUSIONS

Our study identified and explored student‐related, school‐related, and parent‐related barriers and facilitators of physical activity in students in Lagos State. Our study's findings can help design interventions to increase physical activity among school‐attending adolescents in Lagos, Nigeria.

## AUTHOR CONTRIBUTIONS

**Busola Adebusoye**: conceptualization; data curation; formal analysis; funding acquisition; investigation; methodology; project administration; resources; software; validation; writing – original draft; writing – review & editing. **Jo Leonardi‐Bee**: conceptualization; formal analysis; funding acquisition; investigation; methodology; resources; supervision; validation; writing – review & editing. **Revati Phalkey**: conceptualization; funding acquisition; investigation; methodology; resources; supervision. **Kaushik Chattopadhyay**: conceptualization; formal analysis; funding acquisition; investigation; methodology; resources; supervision; validation; writing – review & editing.

## CONFLICT OF INTEREST

The authors declare no conflict of interest

## ETHICS STATEMENT

The study was conducted according to the guidelines of the Declaration of Helsinki and approved by the University of Nottingham's Faculty of Medicine and Health Sciences Research Ethics committee (429‐1912 and 20 January 2020) and the Lagos State Health Research and Ethics Committee (LREC/06/10/1319 and 28 January 2020).

## TRANSPARENCY STATEMENT

The lead author Busola Adebusoye affirms that this manuscript is an honest, accurate, and transparent account of the study being reported; that no important aspects of the study have been omitted; and that any discrepancies from the study as planned (and, if relevant, registered) have been explained.

## Data Availability

The data that support the findings of this study are available from the corresponding author upon reasonable request.
